# Automatic detection of teeth and dental treatment patterns on dental panoramic radiographs using deep neural networks

**DOI:** 10.1080/20961790.2022.2034714

**Published:** 2022-03-09

**Authors:** Hye-Ran Choi, Thomhert Suprapto Siadari, Jo-Eun Kim, Kyung-Hoe Huh, Won-Jin Yi, Sam-Sun Lee, Min-Suk Heo

**Affiliations:** a Department of Oral and Maxillofacial Radiology and Dental Research Institute, School of Dentistry, Seoul National University, Seoul, Republic of Korea; b Artificial Intelligence Research Centre, Digital Dental Hub Incorporation, Seoul, Republic of Korea; c Department of Oral and Maxillofacial Radiology, Seoul National University Dental Hospital, Seoul, Republic of Korea

**Keywords:** Forensic sciences, forensic odontology, individual identification, disaster victim identification, radiography, deep learning, artificial intelligence

## Abstract

Disaster victim identification issues are especially critical and urgent after a large-scale disaster. The aim of this study was to suggest an automatic detection of natural teeth and dental treatment patterns based on dental panoramic radiographs (DPRs) using deep learning to promote its applicability as human identifiers. A total of 1 638 DPRs, of which the chronological age ranged from 20 to 49 years old, were collected from January 2000 to November 2020. This dataset consisted of natural teeth, prostheses, teeth with root canal treatment, and implants. The detection of natural teeth and dental treatment patterns including the identification of teeth number was done with a pre-trained object detection network which was a convolutional neural network modified by EfficientDet-D3. The objective metrics for the average precision were 99.1% for natural teeth, 80.6% for prostheses, 81.2% for treated root canals, and 96.8% for implants, respectively. The values for the average recall were 99.6%, 84.3%, 89.2%, and 98.1%, in the same order, respectively. This study showed outstanding performance of convolutional neural network using dental panoramic radiographs in automatically identifying teeth number and detecting natural teeth, prostheses, treated root canals, and implants.

## Key points

It is useful to use dental panoramic radiographs to perform the disaster victim identification (DVI).

Individual dental treatment patterns could be unique and powerful characteristics as human identifier.

Deep learning can automatically detect teeth and dental treatment patterns with favourable accuracy.

Constructing a big database including detected teeth information would make it possible to fully automate entire process of DVI.

## Introduction

Catastrophes sometimes cause large-scale death such as in the case of the east Japan earthquake and tsunami in Japan in March 2011 and the Daegu subway fire disaster in Korea in February 2003. Disaster victim identification (DVI) becomes more important when the disaster is more widespread. The identification of forensic victims is a difficult and time-consuming task in a large-scale disaster. The most important element of identification specified by the Federal Bureau of Investigation (FBI) in the United States and the International Criminal Police Organization (INTERPOL) is called the Primary Identifier which includes fingerprints, genes, and teeth. In general, DNA analysis is known to be the most precise and delicate method for DVI. However, it requires specific laboratory equipment, and enough well-preserved DNA sample is a critical prerequisite [1].

On the other hand, the dental panoramic radiograph (DPR) is one of the most popular biometrics used in the process of forensic human identification [2]. Using teeth, which is one of the hardest tissues in the human body, for human identification helps to condense potential candidates or to confirm identification accurately. It is more likely that an individual would leave more than one DPR record during their lifetime because dental care is common. The number of remaining teeth has also increased due to the high-level growth of oral hygiene and a longer life expectancy. According to a previous study, the number and complexity of dental restorations increased with age [3]. Another study found that individuals who received a number of complicated dental treatments were easier to identify compared to individuals who received little or no treatment [4]. The probability that any two persons have the exact same dental condition during their whole life is extremely rare [5]. For this reason, individual dental treatment patterns are more diversified which can be unique and powerful characteristics that represent an individual.

Among the available radiographs, the DPR provides useful diagnostic information for the patient as a tomography that shows the maxilla, mandible, and facial structures as one continuous radiographic image [6]. DPR is a reliable tool for DVI because it is periodically taken and updated [7]. In recent years, artificial intelligence (AI) can play an important role in the types of image processing that are cumbersome or time-consuming for the observer [8]. The object detection, image classification, and pattern recognition using deep learning have developed rapidly and are being applied to medical and dental imaging diagnosis [9]. The convolutional neural network (CNN) is a method that is prominent in the image domain, and is particularly useful for finding patterns for image recognition [10] including radiological applications in general medical fields [10,11].

At the present time, there are some existing studies comparing DPR images themselves or individually identifying teeth number or restorations using deep learning [12–14]. To date, few studies have performed a comprehensive detection of all existing dental treatment patterns including teeth number identification as a basis for DVI using a deep neural network. The aim of this study was to develop a model that has not yet been investigated in the field of forensic science. This study aimed to propose a deep learning system that can automatically detect natural teeth and dental treatment patterns and to evaluate its accuracy if it can be potentially used for human identification.

## Materials and methods

### Dataset and annotation

A total of 1 638 DPRs were randomly selected and retrospectively reviewed after de-identifying from the picture archiving and communication system (PACS) at Seoul National University Dental Hospital. The radiographic images of each patient were obtained using a panoramic radiography OP-100 (Imaging Instrumentarium, Tuusula, Finland) and RayScan alpha-P (Ray, Gyeonggi, Korea). The DPRs were collected from January 2000 to November 2020 for the purpose of dental treatment or diagnosis. The chronological age of the subjects ranged from 20 to 49 years old. The exclusion criteria were as follows:

- Primary and mixed dentition
- Fully or partially edentulous dentition
- Patients who had extracted one of the premolar teeth for the purpose of orthodontic treatment
- Impacted teeth other than the third molars
- Image with severe noise, haziness, or distortion

**Figure 1.**
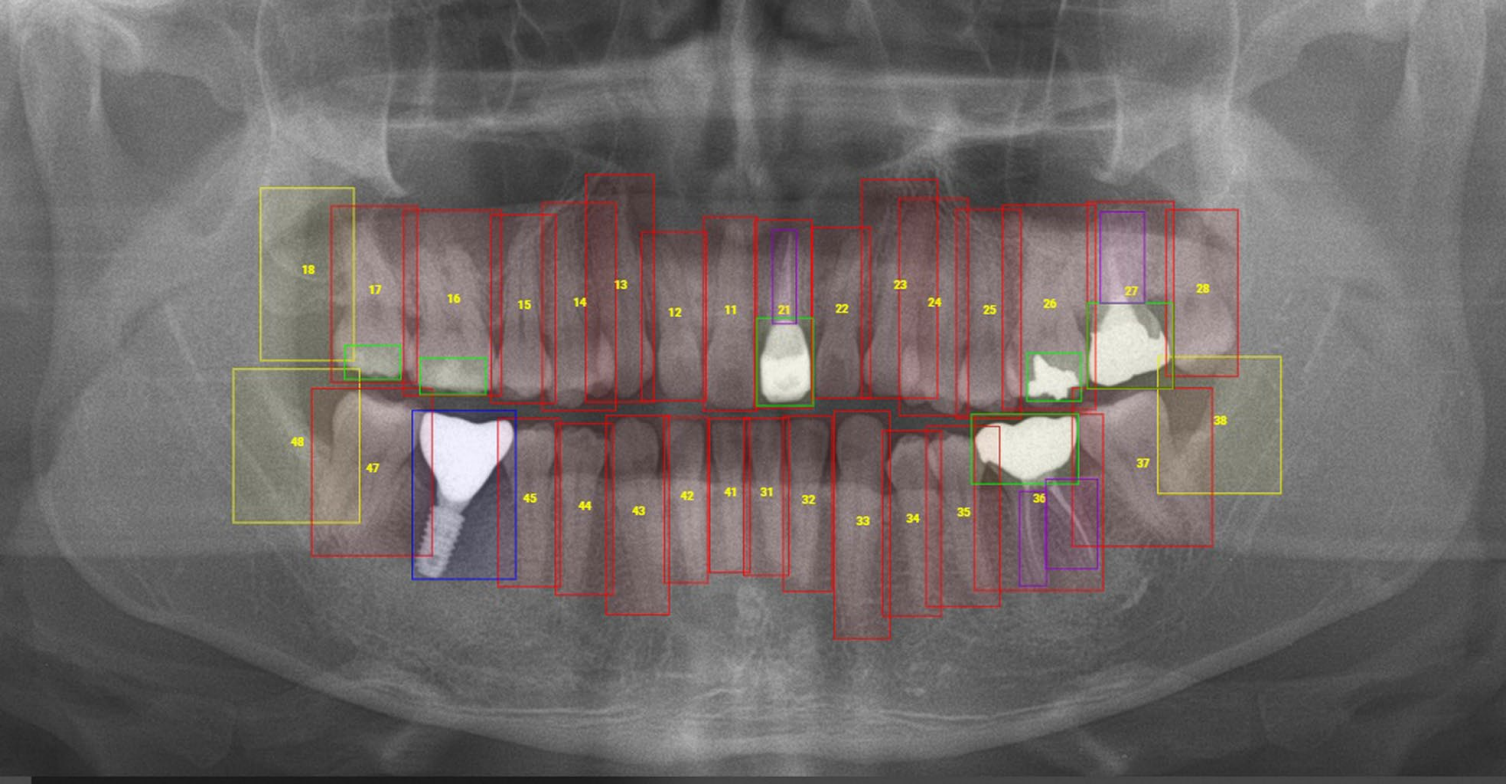
Annotation results using labeling tool for training teeth and dental treatment patterns by the developed convolutional neural network. Red, present of natural teeth and numbering; yellow, lost natural teeth and numbering; lime green, prostheses; purple, treated root canals; blue, implants.

The annotation work was performed on all teeth and dental treatment patterns on the DPRs using bounding box according to four annotation categories with five colours (Figure 1). The categories and coding colours were as follows:

- Natural teeth and numbering (red when present and yellow when lost)
- Prostheses (lime green)
- Treated root canals (purple)
- Implants (blue)

This study defined categories of natural teeth, prostheses, implants, and treated root canals as follows:

- Natural teeth were in a pure state, for example, they had not undergone any treatment.
- Prostheses were defined as the parts that were directly or indirectly restored among the overall shape of teeth. If there was more than one restored region in a tooth, the deep neural network was designed to determine that this tooth was classified as this category.
- Treated root canals referred to the part of roots that shows traces of root canal treatment.
- Implants were classified into an independent category distinct from prostheses. It was defined as a category of implants if an implant fixture was observed alone or an implant fixture with a crown was observed in a certain tooth location.

**Figure 2.**
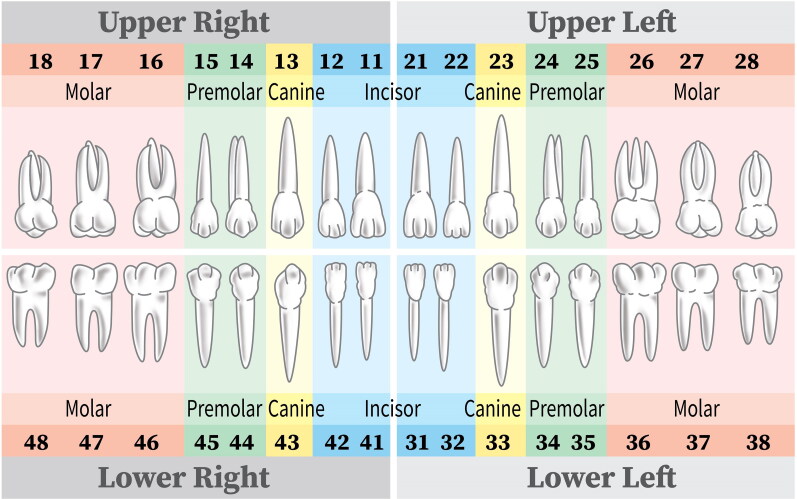
Teeth numbering system proposed by the World Dental Federation.

Natural teeth labeling includes the marks of teeth number simultaneously. The World Dental Federation notation is used to carry out teeth numbering annotation (Figure 2). If a tooth was lost in any position, annotation was conducted at the likely location of the original tooth was predicted. One oral and maxillofacial radiologist manually conducted annotation task consistently using a fully web-browser based labeling system developed by Digital Dental Hub (Seoul, Korea). The types of label export for administration were JSON (JavaScript Object Notation). The cross-check was also performed after all annotation work was completed.

The DPRs were randomly separated into a training set (60%), a validation set (20%), and a test set (20%). Table 1 shows the comprehensive composition of the dataset description used in each procedure for the deep learning architecture. One DPR had combinations of determined categories for all positions of the dentition. The training set was used for the learning of the image patterns; the validation set was used for verification of the training task, and the test set was used to evaluate the final trained model.

**Table 1.** The number of dental panoramic radiographs used in the network.

Category	Training	Validation	Test
Natural teeth	971	359	308
Prostheses	943	351	268
Treated root canals	694	259	215
Implants	271	93	80

### Network architectures and training strategies

Motivated by the recent progress of object detection using deep learning, it was a CNN-based object detection network named EfficientDet for the teeth and treatment pattern analysis on DPRs. EfficientDet was an object detection model that used EfficientNet as the backbone network, a bi-directional feature pyramid network (Bi-FPN), and a compound scaling rule [15,16]. EfficientNet was the CNN-based network that did the compound scaling to scale three subjects which were the depth (number of layers), width (number of channels), and image resolution. Bi-FPN incorporated the multi-level feature fusion that enabled information to flow in both the top-down and bottom-up directions while using regular and efficient connections. Compound scaling simply referred to scaling up all dimensions such as the backbone, input image size, network width and depth at the same time to achieve maximum performance during training. In this study, the EfficientDet-D3 was utilised with the backbone EfficientNet-B3. The EfficientDet-D3 took an input size of 896 × 896 and used the backbone network of EfficientNet-B3 which had a channel multiplier of 1.2 and a depth multiplier of 1.4. The Bi-FPN had 160 channels and six layers, and there were four layers in box and class prediction networks.

**Figure 3.**
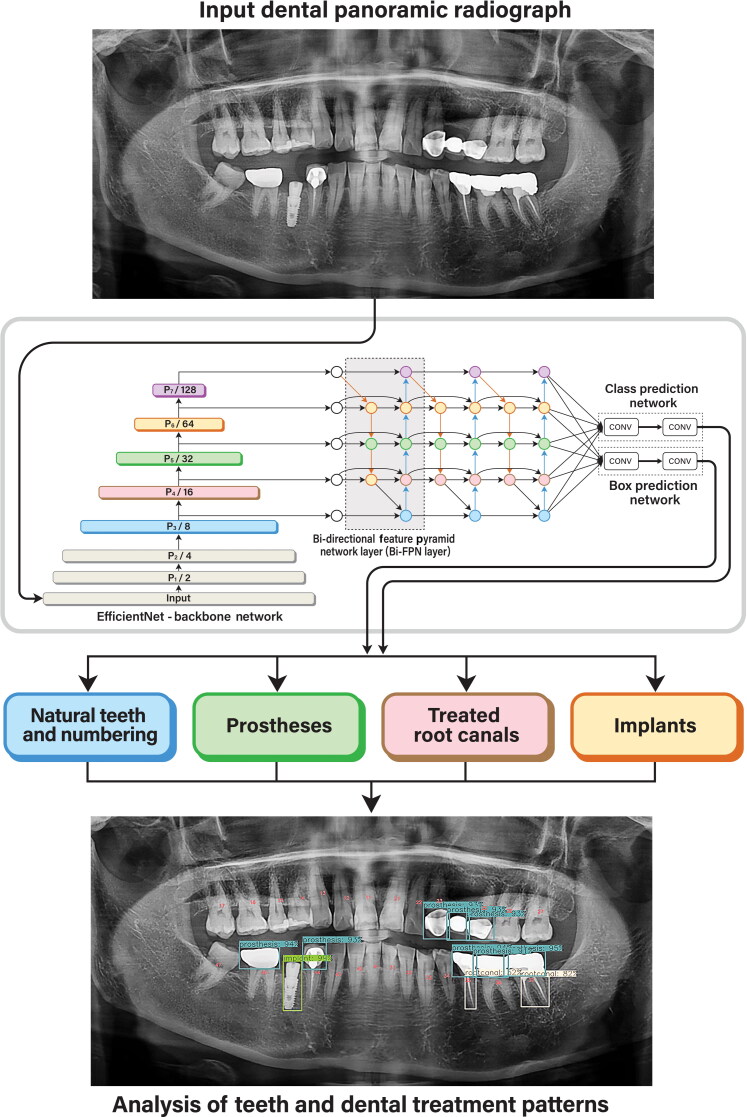
Study flow with the network architecture.

The network architecture was designed properly to ensemble the subset networks for the detection of teeth and dental treatment patterns (Figure 3). Given the input DPR image, each network was trained individually. During the inference stage, the category was determined through the class prediction network. The detection result was finally derived as one of the following four types: natural teeth and their numbering, prostheses, treated root canals, and implants. Particularly, the tooth number was detected only where the tooth was present. No detection was performed for the area of a missing teeth.

The object detection in each category was trained individually using the Focal Loss. The network was trained with a total of 270 iterations at a learning rate of 0.0001, a batch size of 4 and Adaptive Moment Estimation (Adam) optimiser. The experiments were implemented using the Python (Python Software Foundation, Wilmington, DE, USA) language with Pytorch (Facebook, Menlo Park, CA, USA) libraries and conducted on an Quadro RTX 8000 48GB (Nvidia Corporation, Santa Clara, CA, USA) with the Ubuntu OS (Canonical Ltd., London, UK) and Pytorch Frameworks [17]. During the training, the input images were resized into 896 × 896. The data augmentation was performed using the Albumentations library during the training to increase the amount of data for deep learning by modifying the images including the flipping, manipulation, and contrast normalisation of the images [18]. In the concrete, the images were flipped horizontally and vertically, and transformed by manipulation of the brightness, sharpness, and blur.

### Evaluation of detection performance

The performance of the network was evaluated using the standard of average precision (AP) and average recall (AR) under the intersection over union (IoU). A true positive is considered only for prediction scores over 50% and an IoU with a threshold value of 0.5. The values for the AP and AR were calculated under two IoU threshold values of 0.5 and 0.75 for each location of natural tooth number.

This study adopted the method of interpolating the precision at a certain level of recall to calculate the value for AP so that the precision–recall curve is monotonic. The interpolated curve was defined in the interval of [0, 1]. The value for the AP was calculated by sampling the interpolated precision at the *N* reference recall values. The N-Point interpolation was used to choose the levels of recall. The value for the AP was finally calculated as:

$$AP=\;\frac{1}{N}\sum_{n=1}^{N}{Pr}_{\mathrm{interp}}(R_{r}(n))\;$$

where ${Pr}_{\mathrm{interp}}$ is interpolated precision and $R_{r}(n)$ is the set reference recall values.

Otherwise, the value for the AR was calculated as follows:

$$AR=\frac{1}{O}\;\sum_{o=1}^{O}\underset{k|{\mathrm{Pr}}_{t(o)}(\tau (k))>0}{\max}\left\{{Rc}_{t(o)}(\tau (k))\right\}\;$$

where *o* is an IoU overlapped between the ground truth and the prediction bounding box, ${Pr}_{t(o)}(\tau (k))\;$and ${Rc}_{t(o)}(\tau (k))$ are the precision and recall points for a confidence score τ(k) given the IoU threshold *t*(*o*), respectively.

## Results and discussions

### Overall performance

Table 2 shows the overall detection performances of the model.

**Table 2.** Average precision and recall values under a threshold value of the intersection over union of 0.5 for detection performance of natural teeth and dental treatment patterns.

Category	Average precision (%)	Average recall (%)
Natural teeth	99.1	99.6
Prostheses	80.6	84.3
Treated root canals	81.2	89.2
Implants	96.8	98.1

### Natural teeth numbering detection

**Figure 4.**
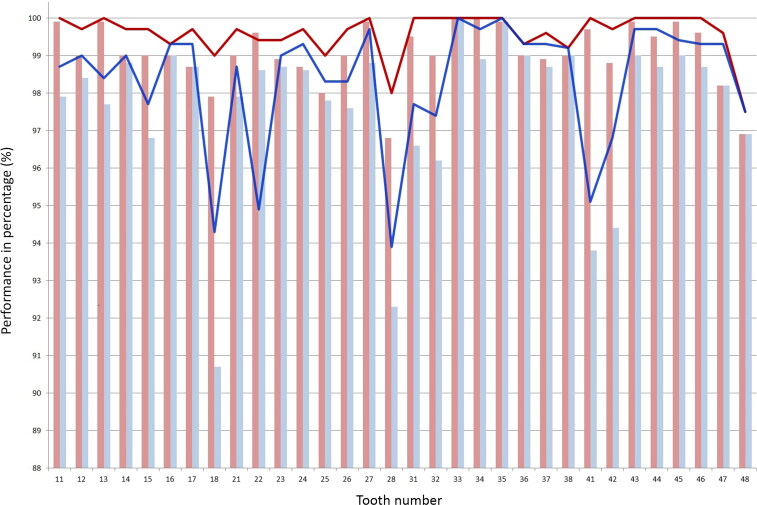
Average precision (AP) and average recall (AR) values under two threshold values of the intersection over union (IoU) for the detection performance of natural teeth numbering. The bar graph shows AP values for IoU threshold values of 0.5 (red) and 0.75 (blue) in that order. The line graph shows the AR values for IoU threshold values of 0.5 (red) and 0.75 (blue) in the similar order from the top.

The detection performance of natural teeth numbering is shown in Figure 4. Slightly lower values for the AP and AR were derived for the location of the mandibular anterior teeth (#31, 32, 41, and 42) and both maxillary third molars (#18, 28) compared to the other values. Additionally, the AR values were low under an IoU threshold value of 0.75 at the positions of the right mandibular third molar (#48) and the left maxillary second incisor (#22).

It was useful to use DPRs to perform the DVI [19,20]. As a basic and core task to fully automate all processes of human identification, this study devised a system that automatically detected dental treatment patterns including teeth using deep learning.

Some studies had utilised radiographs for human identification. Andersen and Wenzel [21,22] used bitewing radiographs to identify victims. There was another study that proposed an identification system that uses image processing to analyse the appearance of teeth in radiographs and to match patterns [23]. On the other hand, there was a study that confirmed the effectiveness of human identification by devising and manually simulating a method to identify individuals by focusing on dentition changes observed on a pair of DPRs [24]. In detail, dental treatment status that followed a chronological step-by-step sequence could demonstrate its usefulness as a key clue for human identification. Nowadays, various models for human identification have been proposed [25–27]. The mainstream themes of existing studies had been to find possible candidates based on the similarity of the image itself or to measure similarities based on various identifiers by extracting certain features or patterns from DPRs [13,27–30]. The latest previous studies using deep learning demonstrated an average accuracy of about 60%–80% [12,14,27,30]; however, this study showed a higher accuracy. Extracting features or performing comparisons on the images could be intuitive and fast, but it might also be affected by several endogenous variables such as the distortion, contrast, and resolution of DPRs.

**Figure 5.**
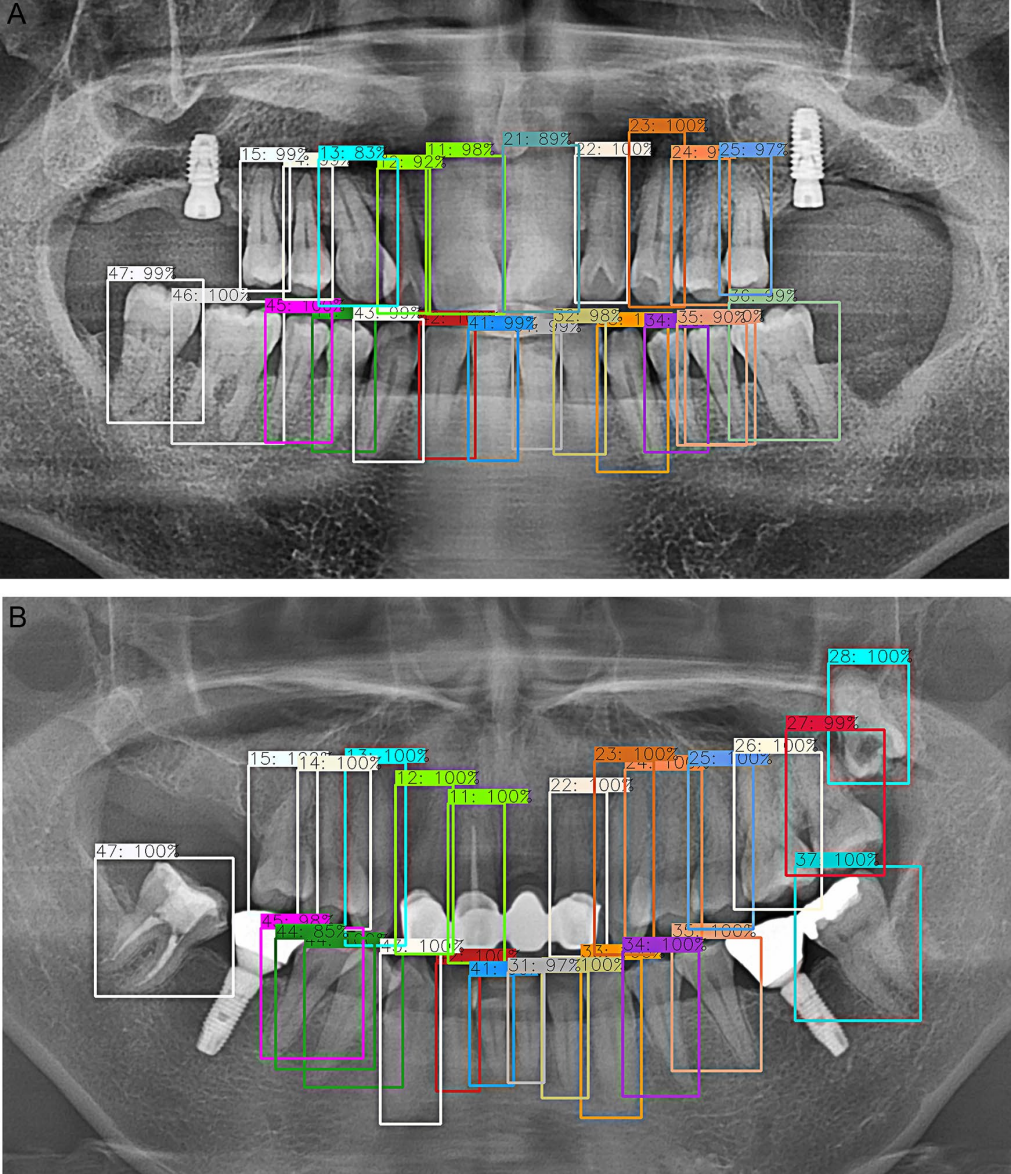
Detection results for natural teeth and their numbering. (A) Overall tooth number was detected with favourable accuracy and without errors although blurring occurred in the upper and lower four incisors. The tooth numbering was performed only where the natural tooth was present. (B) A tooth that was displaced from the occlusal plane, for example, the left upper third impacted molar in this case, showed satisfactory detection performance.

**Figure 6.**
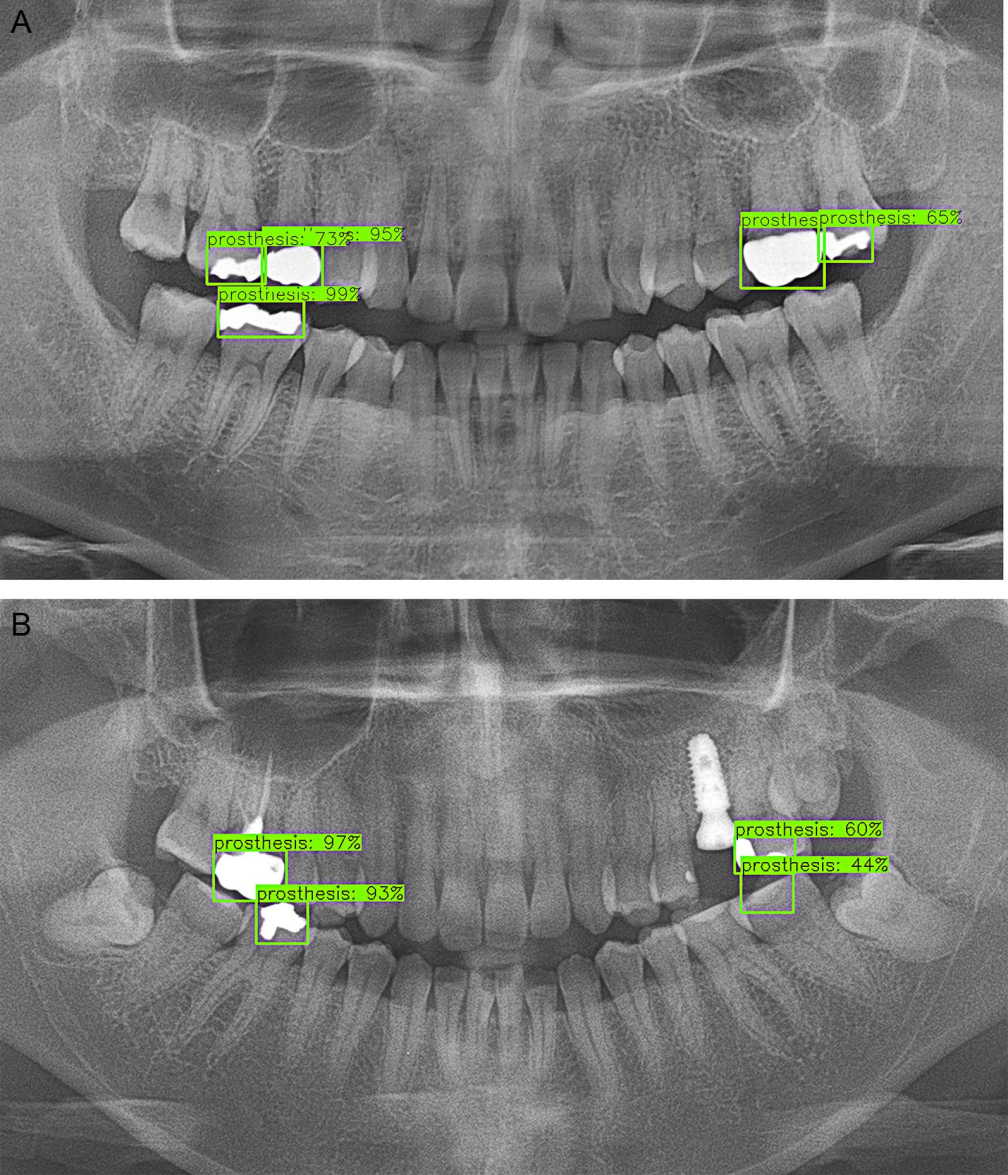
Detection results for prostheses. (A) Prostheses with a high radiopacity were detected with a favourable accuracy. (B) Both the left mandible first molar and second molar were treated with composite resin in this case. The restoration material with a low radiopacity did not achieve a satisfactory outcome. The left mandible first molar was not even detected.

**Figure 7.**
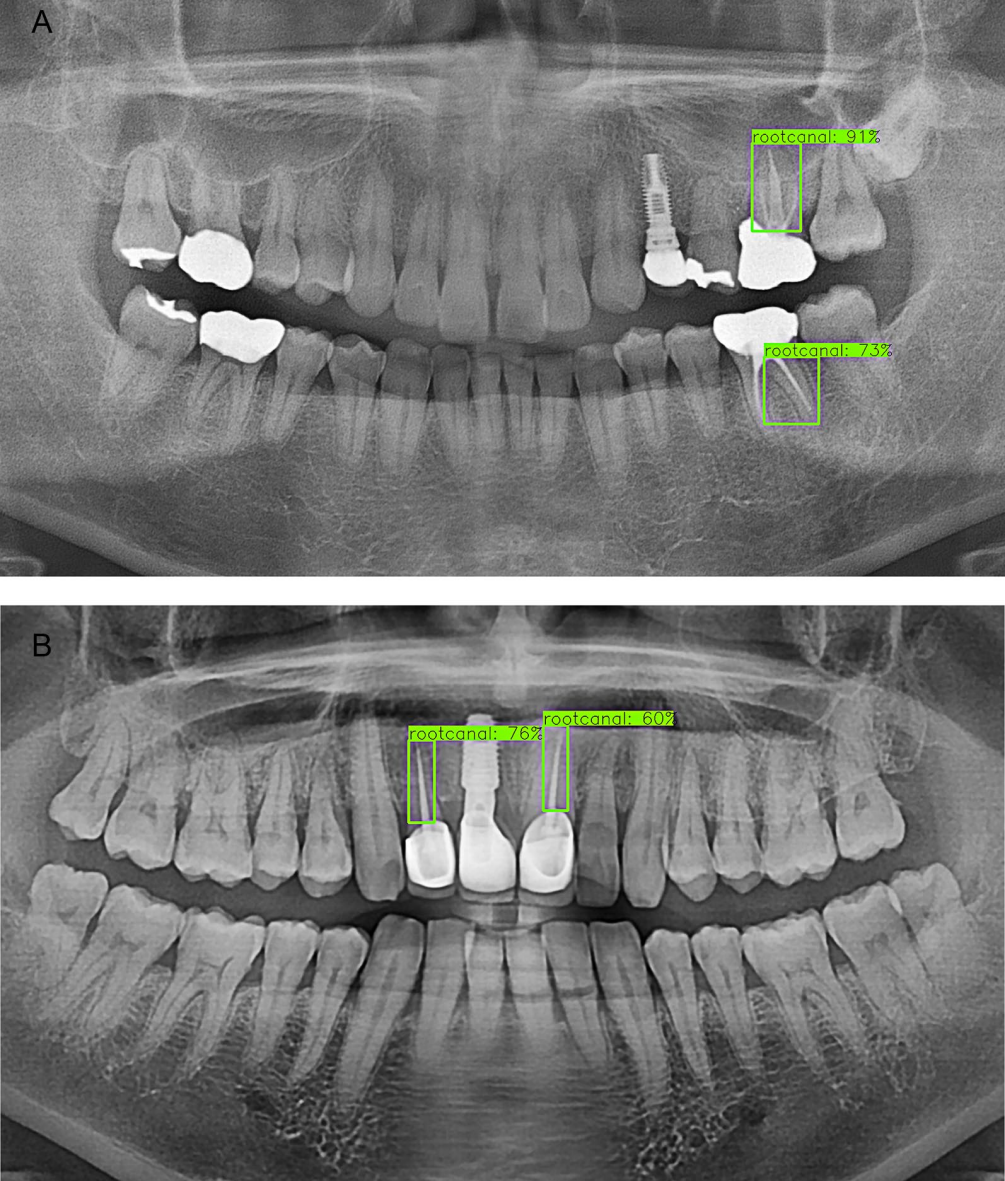
Detection results for treated root canals. (A) The treated root canals that appeared to be adjacent to each other were detected well, but some canals clearly separated by the root were not detected in some cases. (B) The treated root canals in the maxillary anterior region showed a low accuracy.

**Figure 8.**
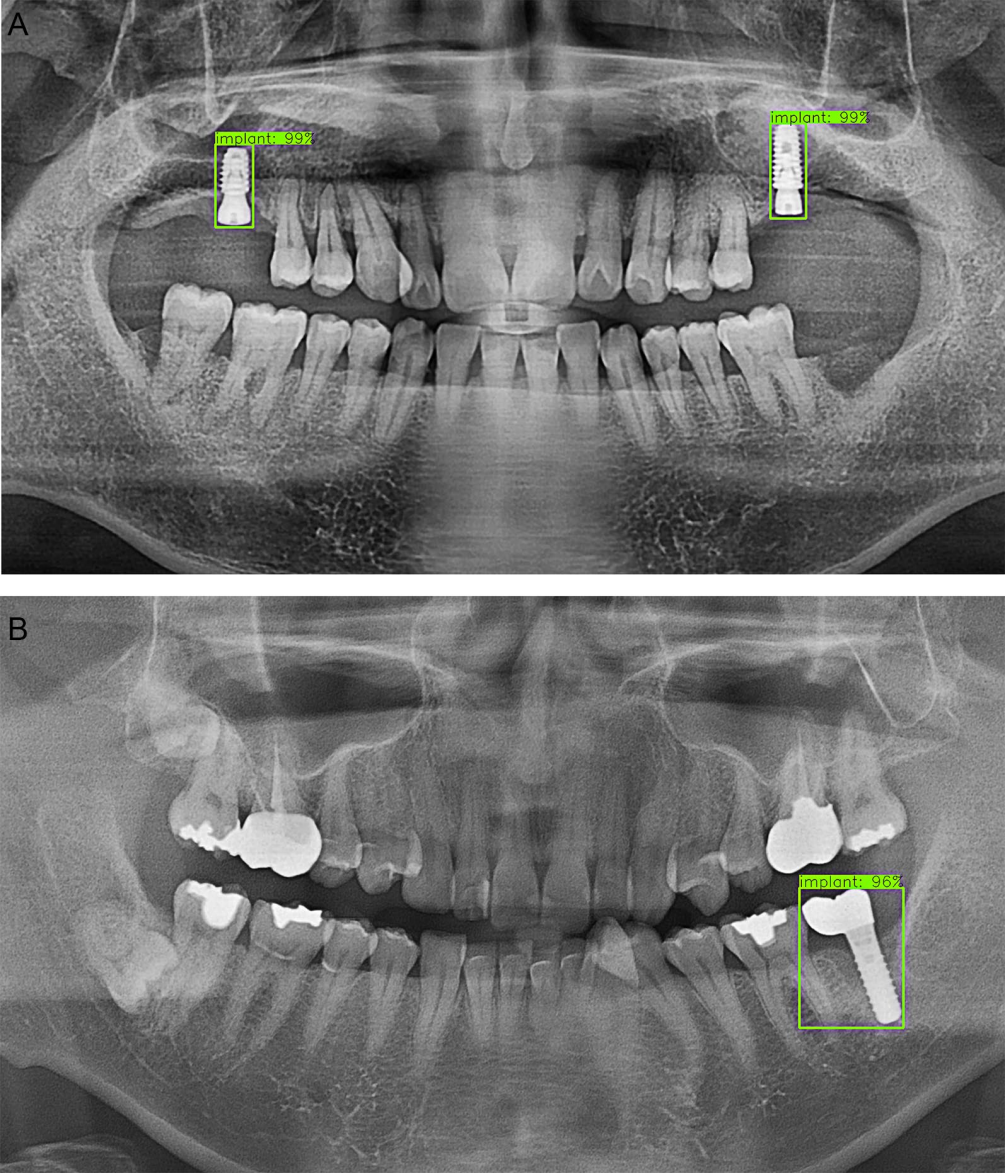
Detection results for implants. (A) An implant was generally detected with high accuracy in a case which was connected with a healing abutment or in a submerged case. (B) The implant connected with the upper prosthetic crown was also well detected.

In this study, a favourable detection performance was shown for natural teeth and implants (Figures 5–8). Natural teeth achieved a favourable outcome in detection where the moderate crowding existed, especially in the anterior part of the mandible. The case in which the upper wisdom tooth or the left maxillary third molar was impacted and displaced from the occlusal plane also showed satisfactory accuracy (Figure 5). First of all, it seemed because the positions of the teeth were consistently aligned in a sufficient sample size and the morphological parts of the implants maintained overall coherent patterns (Figure 8).

However, the performance was rather low when detecting prostheses and treated root canals. One of the reasons seemed that the detection performance was lower when various categories of treatment status were integrated into one broad category, such as in the case of treated root canals or prostheses. For example, there were many different types of treatments in prostheses. Depending on the shape, there were crowns, inlays, bridges, restorative fillings, and so on. On the other hand, gold, metal, porcelain fused to metal (PFM), ceramic, composite resin, etc. were used as materials for the prosthetic treatments. Prostheses with a high radiopacity, such as gold crowns and gold inlays, were detected well, but teeth treated with composite resin, which had a low radiopacity, did not achieve a satisfactory outcome. Both the left mandible first molar and second molar were filled with resin (Figure 6). By the way, only the left mandible first molar was detected with low accuracy and the left mandible first molar was not detected at all in this case. Root canals would have also existed in multiple ways depending on the shape of the tooth. They could be seen in various ways on DPRs. In this study, there were cases where only a part of the treated root canals was detected, especially in the molar area with more than two roots. In the maxillary anterior region, treated root canals were detected with a low accuracy (Figure 7). It was presumed that this was because horizontal distortion occurs more in the middle of the DPR image.

The proposed model in this study generally achieved the promising results with acceptable values for average precision and recall. However, there were limitations that the detection performance did not exceed 90% in some cases such as prostheses and treated root canals. The annotation was labour-intensive and time-consuming task. If more annotated data for training were to be collected with plentiful combinations of dental treatment patterns, the extended model would show more accurate detection performance. On the other hand, the interobserver variability could not be evaluated because only one radiologist annotated the overall data.

As a preliminary study, this study tried to construct a basis for fully automated forensic process including large-scale processing of identification of teeth and their treatment status in the DPRs using deep learning. The result of this study would be used to suggest a method to find an effective way to preemptively eliminate some unexplained discrepancies [4,5,31] that were counter-chronological in comparison to the images themselves. In other words, teeth would have the characteristic of irreversibility in which they could not return to their former state once treated; this could be considered as an advantage of application when performing human identification in a large database in an anonymous DPR. The four categories detected in this study could be aligned on a chronological sequence. For example, if dental caries occurred on natural teeth, restorative treatment could be performed. If secondary caries occurred on this restored tooth, the root canal treatment could be carried out subsequently. If the tooth which had received root canal treatment deteriorated due to periodontitis, tooth would be extracted and an implant might be placed on the extracted site. In fact, this order cannot be reversed physically.

In future studies, the performance could be improved if the quantity and quality of data are enhanced and the training process becomes more sophisticated. It would be needed to collect as much variety of samples as possible in the data collection phase. For efficient learning, samples per training item should be allocated as evenly and diversely as possible. Moreover, if training data for the particularly low part of detection performance are collected intensively for additional training, the performance could also be improved. In addition, if the method of data augmentation is applied in more varying aspects, the issue related to the insufficient amount of data could be overcome and resolved. On the other hand, the results of this study could be extended and applied by segmenting the categories for detection. In dental treatment patterns, for example, prostheses were subdivided into the part of indirect restoration and the part of direct restoration among the overall shape of teeth. These subdivisions of teeth might also include the degree of fracture when a natural tooth was damaged, whether missing teeth existed and types of impactions of wisdom teeth existed. It would effectively contribute to the embodiment of the unique and powerful information of digitised patterns which represents an individual by designing a sophisticated tooth grading system.

## Conclusion

This study showed outstanding performance using CNNs in automatically identifying teeth number and detecting natural teeth, prostheses, treated root canals, and implants. It could be possible to fully automate the entire process of DVI from adding an automated calculation of the detection information and constructing a big database of DPRs to effectively reduce the candidate population in human identification to save massive time and effort.

## Authors’ contributions

Hye-Ran Choi and Thomhert Suprapto Siadari participated in the data collection and formal analysis and prepared the manuscript; Jo-Eun Kim participated in the design of the study and reviewed the manuscript; Kyung-Hoe Huh, Won-Jin Yi, and Sam-Sun Lee provided the resources of the study and performed its coordination; Min-Suk Heo conceived of the study, and managed the project administration and helped to review and edit the manuscript. All the authors contributed to the final text and approved it.
